# Comparative study of physicochemical, nutritional, phytochemical, and sensory properties of bread with plantain and soy flours partly replacing wheat flour

**DOI:** 10.1002/fsn3.2907

**Published:** 2022-04-27

**Authors:** Patchimaporn Udomkun, Cargele Masso, Rony Swennen, Sebastian Romuli, Bhundit Innawong, Apollin Fotso Kuate, Pamela Eloho Akin‐Idowu, Amos Alakonya, Bernard Vanlauwe

**Affiliations:** ^1^ International Institute of Tropical Agriculture (IITA) Bujumbura Burundi; ^2^ International Institute of Tropical Agriculture (IITA) Yaoundé Cameroon; ^3^ International Institute of Tropical Agriculture (IITA) Kampala Uganda; ^4^ Department of Biosystems KU Leuven Heverlee Belgium; ^5^ Institute of Agricultural Engineering, Tropics and Subtropics Group University of Hohenheim Stuttgart Germany; ^6^ Department of Food Technology Faculty of Engineering and Industrial Technology Silpakorn University Nakhon Pathom Thailand; ^7^ Biotechnology Unit National Horticultural Research Institute (NIHORT) Ibadan Nigeria; ^8^ International Maize and Wheat Improvement Center (CIMMYT) Texcoco Mexico; ^9^ International Institute of Tropical Agriculture (IITA) Nairobi Kenya

**Keywords:** bioactive compounds, bread, composite flour, consumer acceptance, glycemic index, nutritional value

## Abstract

Plantain flour (PLF) and soy flour (SF) were used to substitute wheat flour (10% and 20% w/w) in composite bread. Physicochemical, phytochemical, and sensory properties were investigated. Partial substitution by PLF significantly increased (*p* < .05) starch, amylose, ascorbic acid, and potassium content in bread samples. In contrast, a significant improvement (*p* < .05) in protein, fat, amylopectin, and calcium content was observed with SF substitution. Composite bread with PLF and SF together lowered the hydrolysis index (HI) and glycemic index (GI) as compared with whole wheat flour. The molar phytate to minerals (iron, zinc, and calcium) ratio in all composite loaves was lower than reported critical values, except for phytate to iron. Significant differences (*p* < .05) were found in color, specific volume, and texture characteristics of loaves made from partial substitution with PLF and SF. Sensory evaluation revealed that bread with 10% PLF exhibited better scores for appearance and willingness to pay than the control. In contrast, SF negatively affected (*p* < .05) the appearance, texture, color, overall acceptance, and willingness to pay. The trade‐off analysis indicated that PLF can be utilized to produce bread that meets consumers' demands, while incorporating SF as an alternative high‐nutrient density bread will be beneficial to health.

## Graphical abstract

The level of antinutrients in composite breads was higher than control sample. Substitution of plantain or soy flour at high level could negatively influence physical properties of bread. Using plantain substitute can be a more viable option, although soy might have a higher nutritional gain. Substitution of wheat flour by plantain or soy flour improved nutritional properties of bread. Bread with plantain and soy as substitutes for wheat flour reduced HI and GI values.

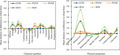

## 1 INTRODUCTION

Bread is a basic staple food in the world and commonly made of wheat flour that contains much protein known as gluten, which is responsible for texture and volume (Gu et al., 2022). Wheat bread is well‐known as a source of calories and complex carbohydrates (Flander et al., 2007; Gomez et al., 2008), but has low micronutrient content, especially of essential amino acids (lysine and threonine), vitamins, and minerals (Turfani et al., 2017; Villarino et al., 2015). In addition, some people are intolerant to glutens of wheat and other related cereals like oats, rye, and barley. This intolerance leads to celiac disease and can cause severe inflammation and mucosal damage of the small intestine (Gutowski et al., 2020; Scherf, et al., 2019). Apart from the wheat bread's low nutritional value, nonwheat‐producing, low‐ and medium‐income countries face the high cost of wheat flour importation. This reduces national resources allocated to food production and, in some cases, increases public debt (Abass et al., 2016). As a result, many efforts emphasize the use of composite flour from indigenous crops such as roots, tubers, cereals, and legumes to partly replace wheat flour to develop nutritionally rich functional foods (Benayad et al., 2021; Chisenga et al., 2020; Dudu et al., 2020; Kotsiou et al., 2022; Nyembwe et al., 2018; Ribotta et al., 2004; Shittu et al., 2007).

Plantains are a multipurpose crop and rich in various bioactive compounds such as dietary fiber, resistant starch, minerals, vitamins, and antioxidants (Amah et al., 2019, 2020; Sojinu et al., 2021; Udomkun et al., 2020, 2021) that have a positive effect on human health (Beara et al., 2012; Roberts et al. 2013). However, the availability and concentration of these compounds vary according to cultivar, ripening stage, growing location, climate, agricultural practices, and processing methods (Udomkun et al., 2020). With approximately 60% of worldwide plantain production, the main plantain‐producing regions are in Central (42%) and West Africa (40%) (FAOSTAT, 2021). Recently, unripe plantain has been used to produce a flour that can be blended with common flours (e.g., wheat, maize, rice, etc.) or ingredients (e.g., protein) in bread (Juarez‐Garcia et al., 2006), pasta (Patiño‐Rodríguez et al., 2019), and snacks (Flores‐Silva et al., 2017). Likewise, Patiño‐Rodríguez et al. (2019) mentioned that unripe plantain flour rarely affects the sensory characteristics of blended flours, hence consumers accept products with this flour.

One of the most important legumes used to raise the nutritional value is soybean. Soybean is a functional food and contains large amounts of protein (38%–40%), fat (18%–20%), and lysine (5%–6%), which have great potential in overcoming protein–calorie malnutrition (Dhingra & Jood, 2001). It is also a rich source of complex carbohydrates, soluble fiber, micronutrients, minerals (Qin et al., 2022), and phytoestrogens (isoflavones) (Hariri et al., 2021). Soy isoflavones are bioactive molecules, which have been hypothesized to have the ability to protect against coronary vascular disease (Hariri et al., 2021; Kim et al., 2022). In addition, several studies have shown the potential health benefits of soybean such as in reducing the risk of colorectal (Yang et al., 2009), prostate, and breast cancer (Shu et al., 2009), controlling blood sugar, and relieving symptoms of several digestive disorders (Hariri et al., 2021). Moreover, soybean has some attractive properties such as high water‐holding capacity, foaming capacity, and tenderizing effect (Nilufer‐Erdill et al., 2012). Thus, incorporating soy flour into bread and improving nutritional value might positively affect the structure and sensory characteristics of finished products.

To completely replace wheat flour with nongluten flours is a big challenge for food technology, given the unique role of gluten in yeast‐leavened baked goods and bread making. The total absence of gluten highly influences dough rheology and elasticity compared to wheat dough (Cappelli et al., 2020). The lack of gluten also leads to bread with a poor texture and color, low specific volume, unsatisfying taste, and other unwanted attributes (Cappelli et al., 2020; Paciulli et al., 2018). Moreover, Houben et al. (2012) showed that bread without gluten resulted in a short shelf‐life, probably because gluten lacks the viscoelastic network. Partial replacement of wheat flour by flour from other crops, without adversely influencing the consumers' acceptability, will be a welcome development for food producers who cannot afford ingredients for gluten‐free bread production. The production of composite flours using various crops for confectionary and bakery products has been presented in many studies, among which are wheat‐legume composite flours (Benayad et al., 2021; Serventi et al., 2018) and wheat‐root/tuber crops composite flours (Amandikwa et al., 2015; Chisenga et al., 2020; Jensen et al., 2015). However, the impact of unripe plantain flour and soy flour, alone and in combination on the composite bread quality is ambiguous. To be accepted by consumers, the inclusion of indigenous flours into wheat flour up to about 20% (w/w) has been recommended for composite bread making (Adebayo‐Oyetoro et al., 2016; Shittu et al., 2007). Therefore, this study aimed to assess the effect of partially incorporating different levels (10% and 20% w/w) of plantain and soybean flours on the nutritional, phytochemical, physical, organoleptic, and trade‐off characteristics of whole wheat bread.

## 2 MATERIALS AND METHODS

### 2.1 Preparation of plantain and soy flours

Plantains (cv. Mbouroukou 3) with deep green undamaged fruits were purchased from a local market in Yaoundé, Cameroon. Fingers from different bunches were randomly picked; fruits were manually cleaned, peeled with a stainless steel knife, and sliced with a dicer into circular discs approximately 3‐mm thick. The plantain slices were evenly spread on a rectangular stainless steel tray and dehydrated by air convection at a temperature of 70 ± 2°C using a laboratory‐scale hot air dryer (LABEC, Laboratory Equipment Pty Ltd.). The drying process was carried out until constant weight was achieved, corresponding to a moisture content of 11.0 ± 0.05% wet basis (w.b.) with water activity at 0.5 ± 0.05. The dried plantain chips were ground with a laboratory grinder (model VMO109, Vita‐Mix Corp.) for 2 min, sieved using 60 mesh sieves (ASTM: 60, 250 μm), then collected and stored in a 250 g aluminum foil bag at room temperature for further bread making. The proximate composition of plantain flour (PLF) was moisture 11.2%, carbohydrate 79.4%, fat 0.6%, fiber 2.6%, protein 3.7%, and ash 2.5%.

Soybeans (cv. Chiangmai 2) were purchased from a local market in Thailand. They were cleaned, sorted, and roasted at 200°C for 40 to 60 min to a moisture content of 11.5 ± 0.5%, then dehulled, allowed to cool, and later ground in a blender. After 30 min of roasting, those seeds were randomly collected every 10 min to measure moisture content. The soybean powder was sieved using 250‐µm mesh size. The proximate composition was moisture 10.8%, carbohydrate 27.1%, fat 15.5%, fiber 4.9%, protein 36.5%, and ash 5.2%.

### 2.2 Bread making

Five kinds of bread were prepared: whole wheat flour (CON) and four in which wheat flour was partly replaced by the following ratios of 10% plantain flour (PLF10), 20% plantain flour (PLF20), 10% soy flour (SF10), and 20% soy flour (SF20). All ingredients were purchased from a supermarket in Thailand (Table 1).

**TABLE 1:** Ingredients for bread making

Raw materials (g/100 g)	CON	PLF10	PLF20	SF10	SF20
Wheat flour	57.8	52.0	46.2	52.0	46.2
Plantain flour	—	5.8	11.6	—	—
Soy flour	—	—	—	5.8	11.6

Note

Every 100 g of all treatments contained 5.8 g of canola oil, 5.8 g of refined sugar, 0.6 g of salt, 1.2 g of dried yeast, and 28.9 g of tap water.

Abbreviations: CON, whole wheat flour; PLF10, 10 g of plantain flour/100 g; PLF20, 20 g of plantain flour/100 g; SF10, 10 g of soy flour/100 g; SF20, 20 g of soy flour/100 g.

All the ingredients were accurately weighed and mixed with a hand mixer (Model 22230‐56, Russell Hobbs) at a low speed for 15 min. After a floor time of 15 min at room temperature, the dough was divided into 200 g portions and then molded manually into loaves. After the final molding, the portions of dough were placed in lightly greased tins, held for another 30 min for final proofing, and then baked in the oven at 200°C for 50 min. The loaves were removed from the tins and cooled to room temperature for 2 h prior to being packed in plastic bags that were sealed to prevent moisture loss. Testing for sensory evaluation was done within 1 day after the loaves had been removed from the oven and within 2 days for bread characteristics. Three batches were produced and analyzed for each bread formulation.

### 2.3 Analysis of bread samples

#### 2.3.1 Proximate analysis

Moisture content was determined using AOAC 934.01 method (1990). The sample was dried at 105°C for 16 h in a draft air system (model UF55, Memmert Oven). The loss in weight was recorded as moisture. Nitrogen content was measured by the Kjeldahl method as described in FOSS (2003). A conversion factor of 6.25 was used to convert total nitrogen to percentage crude protein. Ash content was determined by the method of AOAC 900.02A (1990) that involved burning off moisture and all organic constituents at 600°C in a VULCAN™ furnace (model 3‐1750, Cole‐Parmer). The weight of the residue after incineration was recorded as the ash content.

Fat content was determined by the method of AOAC 960.39 (2005) using the Soxhlet extraction technique (model FOSS Soxtec™ extraction, Sweden). The crude fiber content was determined using fiber extraction equipment (model FOSS Fibertec™ 2010, Sweden). Carbohydrate content was calculated by subtracting the percentages of moisture, crude protein, ash, fat, and crude fiber from 100. All measurements were carried out in triplicate. The caloric value (kcal/100 g) of each loaf was calculated using the coefficients of Atwater (Watt & Merrill, 1963) based on the caloric coefficients corresponding to the contents of protein (4.3 kcal/g), carbohydrate (3.9 kcal/g), and fat (8.8 kcal/g).

#### 2.3.2 Determination of starch and total sugar content

Starch and total sugar content were determined according to Dubois et al. (1956). This involved weighing 0.02 g bread sample into a centrifuge tube with 1 ml ethanol, 2 ml distilled water, and 10 ml hot ethanol. The mixture was vortexed and centrifuged at 537 *g* for 10 min. The supernatant was decanted and used for determining sugar content; the sediment was hydrolyzed with perchloric acid and used to estimate starch content. Phenol sulfuric acid reagent was used for color development, and glucose standards for the estimation of sugar. The absorbance was read with a spectrophotometer (model Genesys G10S) at 490 nm.

#### 2.3.3 Determination of amylose content

Amylose content was determined by iodine binding according to Williams et al. (1970). A sample of 0.1 g was weighed into a 100‐ml conical flask and dissolved with 1 ml of 95% ethanol; the starch was hydrolyzed with 9 ml of 1 mol/L NaOH. The flask was transferred to a water bath, boiled for 10 min, then removed. Distilled water was added to level up to 100 ml. Five milliliter was taken from the 100 ml into another conical flask and 1 ml of acetic acid was pipetted into both flasks plus 2 ml iodine solution to change the color. Distilled water was added to level up to 100 ml and the absorbance was read at 620 nm with the spectrophotometer (model Genesys G10S). Amylopectin content was computed by difference from 100.

#### 2.3.4 Starch hydrolysis index and glycemic index

The method of Hsieh et al. (2017) was modified to determine starch hydrolysis. All samples were dehydrated at 60°C for 40 min and then ground with a blender (Y46, Moulinex). One gram of bread powder was mixed with 20 ml of sodium potassium phosphate buffer (0.05 mol/L, pH 6.5) at 65°C for 30 min and subsequently made up to a final volume of 25 ml with the sodium potassium phosphate buffer. A 25‐ml portion of the starch–α‐amylase mixture, consisting of 1 g sample solution and 0.04 g α‐amylase (Sigma‐Aldrich Chemie GmbH), was dialyzed in a dialysis tubing with a cutoff molecular weight of 12,000 to 14,000 DA (Spectra/Por^®^ molecular porous membrane tubing, Spectrum Laboratories, Inc., Breda, The Netherlands) against 200 ml of deionized water at 37°C. Thereafter, the glucose contents in 1.5 ml of those dialysates at 20, 30, 60, 90, 120, 150, and 180 min were enzymatically investigated with the diagnostic kits (Randox Laboratories Ltd., Crumlin Co.). The hydrolysis index (HI) of bread was calculated using the following Equation (1):

(1) $$\text{HI}=({\text{AUC}}_{\text{sample}}/{\text{AUC}}_{\text{reference}})\times 100$$

where AUC_sample_ is the area under the curve of hydrolyzed bread samples and AUC_reference_ is the area under the curve of the reference food (CON sample) up to 180 min.

In this study, bread made with wheat flour was used as a control to estimate the hydrolysis index (HI = 100). The predicted glycemic index (GI) was calculated using Equation (2):

(2) GI=(0.549×HI)+39.71

#### 2.3.5 Determination of ascorbic acid

Vitamin C was determined by the reduction of an oxidation–reduction indicator dye, 2,6‐dichloroindophenol by ascorbic acid to a colorless solution. About 50 g of bread samples was immediately extracted with 200 ml metaphosphoric–acetic acid solution by blending with a Waring blender for 3 min. The resulting slurry was then filtered and 50 ml of the extract was titrated with the dye solution. The endpoint was noted when analyte appears rose pink in color for more than 5 s.

#### 2.3.6 Mineral analysis

Potassium (K) in bread samples was determined using flame photometer (model 410, Sherwood Scientific Ltd.). Contents of zinc (Zn), iron (Fe), calcium (Ca), and magnesium (Mg) were analyzed using atomic absorption spectrophotometer (AAS) (model 205, Buck Scientific).

#### 2.3.7 Bioactive compounds

Phytate was determined using anion exchange method, following Ma et al. (2005) with a slight modification. Two grams of samples were accurately weighed and then transferred into 100 ml conical flasks. A total of 50 ml of 10% Na_2_SO_4_ (w/v) in 1.2% HCl (v/v) was added. Flasks were capped and shaken vigorously for 2 h on a rotator at room temperature. The supernatant was filtered through qualitative filter paper no. 4. Subsequently, 10 ml of filtered extract was diluted to 30 ml with distilled water after being mixed with 1 ml of 0.75 M NaOH. The diluted sample was passed through a 200–400 mesh AG1‐X8 chloride anion exchange resin (Bio‐Rad Laboratories GmbH). After sample application, the column was washed with 15 ml of distilled water and 20 ml of 0.07 M NaCl solution to remove the inorganic phosphate. Then, the retained phytic acid was eluted with 0.7 M NaCl. A total of 4 ml of the reagent was added into 5 ml of collected eluate and centrifuged at 2500 *g* for 10 min. The absorbance of the supernatant was measured at 500 nm using a spectrophotometer (model Genesys G10S). A calibration curve for the colorimetric method was obtained by using sodium phytate standards (P‐8810 Sigma).

Total polyphenols were extracted according to Singh and Jambunathan (1981) and estimated as tannic acid equivalents according to the Folin–Denis procedure (Swain & Hills, 1959). The mole of phytate and minerals (Fe, Zn, and Ca) was determined by dividing the weight of phytate and minerals with its atomic weight (phytate: 660 g/mol; Fe: 56 g/mol; Zn: 65 g/ mol; Ca: 40 g/mol). The molar ratio between phytate and minerals was obtained after dividing the mole of phytate with the mole of minerals.

#### 2.3.8 Colorimetric measurement

Color of bread crust samples was assessed with a Chroma‐Meter (model CR‐100, Minolta Camera Co., Ltd.) using the CIE L*a*b* color system. Before the measurement, the colorimeter was calibrated with a standard white plate D65 (*Y* = 87.5, *x* = 0.3180, and *y* = 0.3355). The values indicated are L* lightness–darkness, a* redness–greenness, and b* yellowness–blueness of bread crust. The white index (WI) was calculated following Equation (3).

(3) $$\text{WI}=100‐{\left[{\left(100‐L^{*}\right)}^{2}+a^{*2}+b^{*2}\right]}^{1/2}$$

#### 2.3.9 Specific volume measurements

Seed displacement method was used to determine the specific volume (cm^3^/g) of bread samples; the loaf volume per bread weight was calculated according to the AACC approved method (2020).

#### 2.3.10 Texture analysis

A texture analyzer (model TA‐XT2i, Stable Micro System Co., Ltd.) was applied on crust and crumb to describe the changes in textural characteristics of bread samples. Crust hardness was measured on five preselected points by means of a puncture test using a 3‐mm diameter stainless steel probe and a test speed of 2 mm/s. Maximum peak force (N) from the penetration curve was taken as crust hardness. Crumb evaluation was carried out on 10 cubes of 20 × 20 × 20 mm extracted from two central slices using a texture profile analysis (TPA) test. A test was performed with a 35‐mm diameter cylindrical aluminum probe by means of a double compression with a speed of 1 mm/s up to the 75% of the original sample height. The textural parameters considered were hardness (maximum peak force of the first compression cycle, N), cohesiveness (ratio of positive force area during the second compression as compared to that during the first compression area, dimensionless), and chewiness (product of hardness × cohesiveness × springiness, maximum force N) (Bourne, 1978). In addition, the normal stress was defined as the maximum force (N) during the first compression per unit area (m^2^). The normal strain was defined as the distance (m) at the maximum force per sample thickness (m). The texture map was developed from the relationship between normal stress and normal strain.

#### 2.3.11 Consumer acceptability

Bread samples were assessed by 93 untrained Thai consumers from Silpakorn University, Thailand (42 males and 51 females, aged 18 to 58 years, with 26 years average). The panelists were prescreened; only those who consumed bread regularly were invited to participate. A five‐point hedonic scale (5 = *like extremely* and 1 = *dislike extremely*) was used to evaluate the product quality in terms of appearance, texture, flavor, color, taste, general acceptance, and willingness to pay. The test samples were put in plastic bags and labeled with a random three‐digit number. The results for each quality parameter were expressed as an average of the quality scores from all the panelists.

### 2.4 Trade‐off analysis

Trade‐off analysis is an approach to positive analysis that combines simulation modeling tools from the relevant disciplines, including agrifood systems (Antle & Valdivia, 2021). The trade‐off process involves losing one quality, aspect, or amount of something in return for gaining another quality, aspect, or amount. In this study, therefore, the concept of trade‐off analysis was applied to examine how the level of indigenous flours can influence the physicochemical, nutritional, phytochemical, and sensory properties of composite bread. This might help decision processors formulate and evaluate forward decisions to improve the nutritional well‐being/health of the people, especially in developing countries. The trade‐off analysis was conducted by calculating the difference of the values of each indicator of physicochemical properties and consumer acceptance from the respective value of the wheat‐based bread. To compare different properties and indicators, the value of the difference was divided by the mean value of the wheat‐based bread.

### 2.5 Statistical analyses

All analyses were performed in triplicates, except for specific volume and textural properties which were carried out in five replications. Mean values with the standard deviations (SD) were reported. Analysis of variance and Duncan's multiple range test were used. SAS program (Ver. 9.4, SAS Inst.) was used for analysis. *p* Values <.05 were regarded as significant.

## 3 RESULTS AND DISCUSSION

### 3.1 Nutritional properties

Moisture content in all bread samples varied between 20.6% and 21.7% (Table 2). Composite bread with PLF substitution had the lowest protein and fat contents; the highest protein and fat contents were significantly found (*p* < .05) in SF substitution. Specifically, the contents of protein increased by 46.9% and of fat by 23.3% with SF20 as compared with CON. The protein and fat contents of the PLF composite bread decreased with increasing levels of flour substitution. This may have been due to the lower content of protein and fat in PLF which caused these dilutions in the whole wheat flour. A similar finding was reported by Inyang and Asuquo (2016) for PLF composite functional bread. The fiber and ash contents in the composite bread increased with the level of PLF and SF substitution. The high‐fiber content of both PLF and SF composite breads suggests that they would be ideal supplementation for people suffering from noncommunicable diseases such as obesity and diabetes. The lowest content of carbohydrate, starch, and amylose was significantly observed (*p* < .05) in composite bread with SF substitution; bread with PLF substitution showed the highest content. Results also indicated that PLF and SF did not significantly influence (*p* > .05) the caloric value of bread samples when compared to CON.

**TABLE 2:** Nutritional characteristics of control wheat bread and samples with replacement by plantain and soy flours

Quality	Units	CON	PLF10	PLF20	SF10	SF20
Proximate						
Moisture	g/100 g	20.7^a^ (0.2)	20.6^a^ (0.1)	21.4^a^ (0.1)	21.7^a^ (0.2)	21.6^a^ (0.2)
Protein	g/100 g	7.7^c^ (0.1)	6.3^c^ (0.2)	6.1^c^ (0.3)	12.1^b^ (0.1)	14.5^a^ (0.0)
Fat	g/100 g	6.9^b^ (0.0)	6.5^b^ (0.0)	6.0^b^ (0.1)	8.9^a^ (0.0)	9.0^a^ (0.0)
Crude fiber	g/100 g	1.1^c^ (0.3)	1.7^b^ (0.3)	2.0^b^ (0.2)	1.8^b^ (0.3)	2.3^a^ (0.1)
Ash	g/100 g	1.4^c^ (0.1)	1.5^c^ (0.0)	1.9^b^ (0.1)	2.0^b^ (0.0)	2.4^a^ (0.0)
Carbohydrate	g/100 g	62.2^a^ (0.2)	63.4^a^ (0.2)	62.6^a^ (0.0)	53.5^b^ (0.0)	50.2^b^ (0.1)
Starch	g/100 g	54.5^b^ (0.1)	57.8^a^ (0.0)	60.2^a^ (0.0)	48.5^c^ (0.1)	47.7^c^ (0.3)
Total sugar	g/100 g	9.4^a^ (0.1)	7.2^c^ (0.0)	7.4^c^ (0.1)	7.5^c^ (0.1)	8.7^b^ (0.1)
Amylose	g/100 g	40.3^c^ (0.1)	47.5^b^ (0.1)	55.9^a^ (0.1)	25.8^d^ (0.1)	27.9^d^ (0.1)
Amylopectin	g/100 g	59.7^b^ (0.1)	52.5^c^ (0.1)	44.1^d^ (0.1)	74.2^a^ (0.1)	72.1^a^ (0.1)
Caloric value	kcal/100 g	336.4^a^ (0.6)	331.6^a^ (0.8)	323.2^a^ (0.1)	339.0^a^ (0.1)	337.3^a^ (0.1)
Hydrolysis index (HI)	g/100 g	100.0^a^ (0.0)	80.1^b^ (0.5)	68.5^c^ (0.4)	82.2^b^ (0.2)	70.3^c^ (0.3)
Glycemic index (GI)	g/100 g	94.6^a^ (0.0)	83.7^b^ (0.4)	77.3^c^ (0.3)	84.8^b^ (0.3)	78.3^c^ (0.1)
Ascorbic acid	mg/100 g	3.74^c^ (0.00)	5.25^b^ (0.00)	6.50^a^ (0.00)	3.50^c^ (0.00)	3.75^c^ (0.00)
Minerals						
Potassium (K)	mg/100 g	202.8^d^ (1.3)	232.5^bc^ (1.5)	257.6^a^ (1.3)	226.4^c^ (1.6)	240.5^b^ (1.4)
Zinc (Zn)	mg/100 g	1.8^c^ (0.2)	2.2^b^ (0.4)	2.6^a^ (0.2)	2.7^b^ (0.2)	3.1^a^ (0.2)
Iron (Fe)	mg/100 g	3.2^c^ (0.2)	4.5^b^ (0.1)	4.8^a^ (0.3)	4.3^b^ (0.1)	4.7^a^ (0.5)
Calcium (Ca)	mg/100 g	119.6^b^ (1.4)	115.9^c^ (1.4)	112.5^c^ (1.5)	124.2^a^ (1.6)	128.6^a^ (1.5)
Magnesium (Mg)	mg/100 g	115.4^c^ (0.1)	117.9^c^ (0.1)	122.6^b^ (0.2)	124.3^b^ (0.1)	128.9^a^ (0.3)
Phytochemicals						
Phytic acid	mg/100 g	93.8^e^ (1.2)	102.5^d^ (0.9)	124.6^b^ (1.3)	111.6^c^ (0.8)	134.5^a^ (1.5)
Polyphenols	mg/100 g	195.6^d^ (2.1)	221.4^b^ (1.8)	245.6^a^ (1.2)	211.5^c^ (2.0)	228.9^b^ (1.9)

Note

All values show the mean (standard deviation). ^a–e^Values within a row with different letters are significantly different (*p* < .05).

Abbreviations: CON, whole wheat flour; PLF10, 10 g of plantain flour/100 g; PLF20, 20 g of plantain flour/100 g; SF10, 10 g of soy flour/100 g; SF20, 20 g of soy flour/100 g.

The hydrolysis index (HI) value is a proxy of the glycemic index (GI) in healthy subjects (De Angelis et al., 2009). Due to the rapid increase of blood glucose and secretion of insulin, the intake of high GI foods is associated with the occurrence of carbohydrate metabolic disorders and cardiovascular disease (Kaur et al., 2015; Li & Hu, 2022; Ma et al., 2012). When the HI value of CON was assigned at 100, a significant reduction (*p* < .05) of HI and GI values was found in composite bread samples with PLF and SF substitution (Table 2). Although the predicted GI values of all samples were classified as high (>70), this finding showed that partial replacement by PLF20 and SF20 potentially decreased the GI value by 18.3% and 17.2%, respectively. A low level of GI value in composite bread with PLF and SF substitution could be explained by its high fraction of resistant starch (RS) and slowly digestible starch (SDS) (Haque et al., 2020; Olawoye et al., 2020). In addition, Hsieh et al. (2017) reported that the soluble fibers in grain might act as a barrier which interferes with starch hydrolysis, causing low starch degradation of whole grain steamed Chinese buns (*Mantou*). The lower HI in oat bread was related to the presence of soluble fiber β‐glucan (Wolter et al., 2013). Although PLF and SF could contribute to the low GI value, the thermal processing, according to Guillén et al. (2018), might lead to a reduction in RS and SDS during the production of bread, increasing the GI value in composite bread.

The result also showed that partial substitution with PLF10 significantly increased (*p* < .05) ascorbic acid content in composite bread by 28.8% and with PLF20 by 42.5% when compared to CON. There was no significant difference (*p* > .05) in the ascorbic acid content between CON and SF (Table 2). Substituting PLF and SF for wheat significantly improved (*p* < .05) mineral content in composite bread. When the mineral contents are considered, it could be observed that partly substituting PLF and SF for wheat significantly improved (*p* < .05) mineral content in composite bread. With an increasing level of flour substitution, PLF was found to contain higher amounts of K and Fe than CON and SF. These findings could imply that the consumption of composite bread is recommended because it is richer in mineral content. A previous study of Inyang and Asuquo (2016) confirmed that K, Fe, and Mg contents increased with increasing levels of unripe PLF substitution. However, they reported a reduction of Zn in the PLF composite bread samples which is contrary to our findings. A higher amount of minerals (Fe, Zn, Mn, Cu, Ca, and Mg) in SF when compared with wheat flour was demonstrated by Issa Khan et al. (2005).

Antinutrients are special type of phytochemicals which are known to interfere with the uptake of nutrients by the body, causing harm to the biological system (López‐Moreno et al., 2022; Udomkun et al., 2019). Phytic acid has been identified to have beneficial effects for health such as anticarcinogenic and antioxidant properties (Campos‐Vega et al., 2010; Samtiya et al., 2020). However, it can form very stable, insoluble complexes with minerals such as Ca, Zn, and Fe (Bohn et al., 2008) as well chelate amino acids, thereby decreasing mineral and amino acid bioavailability (Kruger et al., 2013). Similarly, polyphenols are beneficial to health, but their interaction with the food metric could affect mineral availability (Ferruzzi et al., 2020) and digestibility of protein and carbohydrate (Oghbaei & Prakash, 2016). In this study, the phytic acid and polyphenols in composite bread significantly increased (*p* < .05) with the rise in PLF and SF substitution (Table 2). The maximum increase of 30.3% in phytic acid and of 20.4% in polyphenols over CON was found in bread containing SF20 and PLF20, respectively. Nissar et al. (2017) indicated that phytate content should be lowered as much as possible, ideally ≤25 mg/100 g or 3% of the phytate‐containing food should be consumed to minimize micronutrient losses. However, the reference daily intake (RDI) value of phytate varies by country; for example, 180 mg RDI/day has been specified for Sweden, whereas UK and USA accept 631–746 mg RDI/day (Nissar et al., 2017). As phytate is heat stable, baking might not degrade it substantially owing to the formation of either insoluble complexes between phytate and other macro‐ and micronutrients, such as phytate–protein and phytate–protein–mineral complexes, or the penta‐ and tetra‐phosphate hydrolyzed products of inositol hexaphosphate (Siddhuraju & Becker, 2001).

**FIGURE 1:**
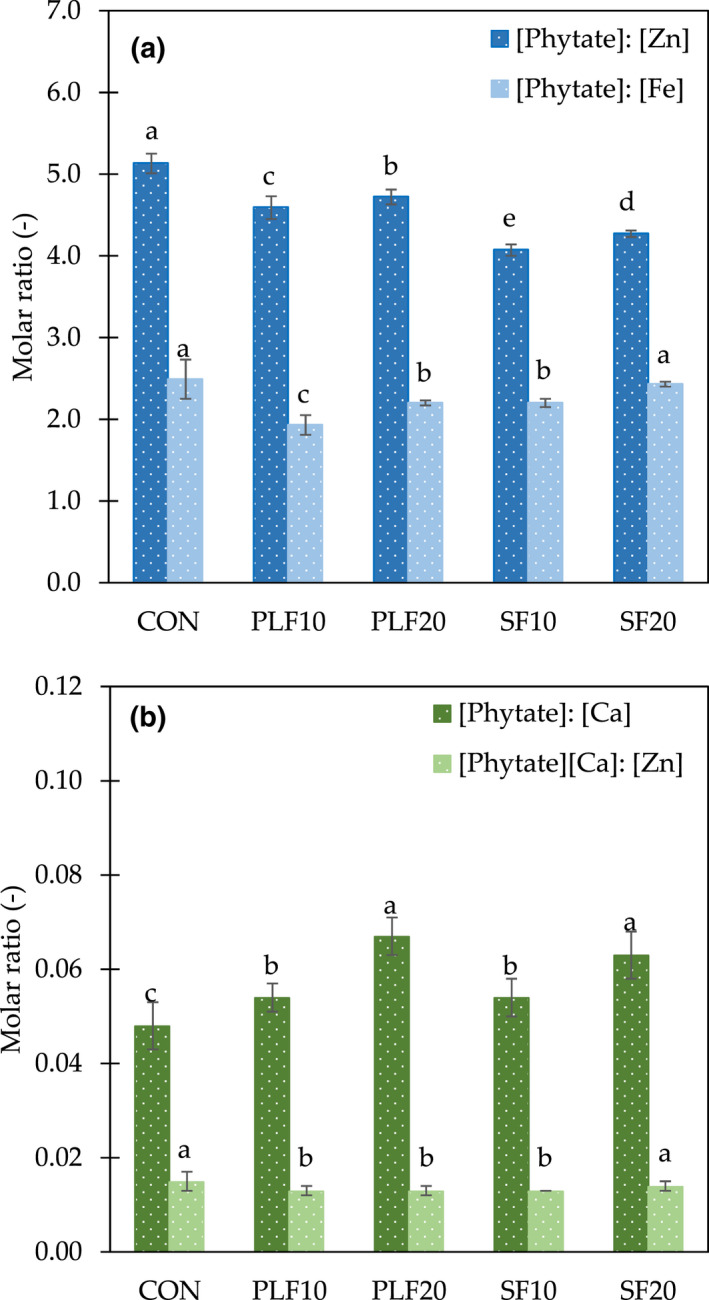
Molar ratio of phytate to minerals of control wheat bread and samples with replacement by plantain and soy flours. (a) [phytate]:[Zn] and [phytate]:[Fe]; (b) [phytate]:[Ca] and [phytate][Ca]:[Zn]^1^. ^1^Unit of [phytate][Ca]:[Zn] is mol/g. ^a–e^Values within a bar graph of each phytate to mineral molar ratio with different letters are significantly different (*p* < .05). CON, whole wheat flour; PLF10, 10 g of plantain flour/100 g; PLF20, 20 g of plantain flour/100 g; SF10, 10 g of soy flour/100 g; SF20, 20 g of soy flour/100 g

Due to the high phytate content in studied bread samples, it is crucial to consider its effect on the bioavailability of essential minerals such as Zn, Fe, and Ca. The results show that the molar ratios of [phytate]:[Zn] and [phytate]:[Fe] were significantly lower (*p* < .05) in composite bread when compared to CON, except for the molar ratio of [phytate]:[Fe] of SF20 which was statistically insignificant (*p* > .05) from CON (Figure 1a). A substitution of SF10 in bread could increase Zn availability by 20.6% when compared to CON; Fe availability approximately increased by 22.5% with PLF10 application. Interestingly, a significant increase (*p* < .05) of [phytate]:[Ca] molar ratios was observed in bread sample, especially at high levels of PLF and SF substitution where that Ca availability decreased by 28.4% with PLF20 substitution and by 23.8% with SF20 (Figure 1b).

When the molar ratio is above 15 for Zn (Ma et al., 2007), 1.0 for Fe (Hurrell, 2004), and 0.17 for Ca (Umeta et al., 2005), the bioavailability of these minerals is inhibited. It could be seen that the phytate to mineral molar ratios of all bread samples were lower than the reported critical values, except [phyate]:[Fe]. Therefore, a significant adverse effect of bread consumption due to low Fe availability should be of concern. In general, phytate inhibition of Fe and Zn bioaccessibility can be minimized by dephosphorylation through thermal or enzymatic means (Ferruzzi et al., 2020). Frontela et al. (2011) reported that the use of sprouting (malting), lactic acid bacteria fermentation or phytase addition could increase Fe and Zn bioavailability in whole grain and refined wheat bread; however, a reduction of Fe and Zn was observed after baking. A study of Garcia‐Mantrana et al. (2015) also showed that the use of *Bifidobacterium pseudocatenulatum* ATCC27919, a phytase producer, as a starter in sourdough could reduce phytate content in whole rye‐wheat mixed bread.

Interestingly, the effect of phytate on the bioavailability of minerals also depends not only on the phytate content but also on the interaction between phytate and minerals (Ma et al., 2007). A finding of Gemede et al. (2015) illustrated that high dietary calcium impairs Zn absorption during high consumption of phytate. Thus, the molar ratio of [phytate][Ca]:[Zn] should be used as an indicator of Zn bioavailability rather than the molar ratio of [phytate]:[Zn] alone (Obah & Amusan, 2009). Adetuyi et al. (2011) indicated that a [phytate][Ca]:[Zn] molar ratio >0.5 will diminish Zn absorption. In this study, the [phytate][Ca]:[Zn] molar ratio of all bread samples was lower than the critical value. In addition, a significantly lower (*p* < .05) amount of [phytate][Ca]:[Zn] molar ratio was found in PLF10, PLF20, and SF10 (Figure 1b).

### 3.2 Physical characteristics

Partial replacement of wheat flour by PLF caused an insignificant change (*p* > .05) in all color values, except b* value, when compared to CON (Table 3). A higher level of PLF caused a slight increase in b* and WI parameters, which indicates more yellow. In contrast, a substitution of SF, especially SF20, resulted in a significant reduction (*p* < .05) of L*, b*, and WI, while the a* value highly increased. Compared to CON, a reduction of WI was observed by 13.5% for SF10 and by 17.0% for SF20. The darker color of bread samples with SF substitution may be due to the presence of brown pigmentation in the SF and Maillard reaction during processing (Ivanovski et al., 2012; Turfani et al., 2017). Similar reduced whiteness of bread crumb was obtained after the addition of fiber from flaxseed by Koca and Anil (2007) and from rice bran by Irakli et al. (2015).

**TABLE 3:** Physical characteristics of control bread and samples with wheat replaced by plantain and soy flours

Quality	Units	CON	PLF10	PLF20	SF10	SF20
Crust color						
Lightness (L*)	—	63.9^a^ (0.4)	64.5^a^ (0.3)	65.2^a^ (0.3)	55.4^b^ (0.2)	53.4^b^ (0.3)
Redness (a*)	—	0.8^c^ (0.1)	1.1^c^ (0.0)	0.9^c^ (0.1)	2.6^b^ (0.3)	4.3^a^ (0.1)
Yellowness (b*)	—	13.4^b^ (0.2)	15.7^a^ (0.2)	16.0^a^ (0.3)	10.8^c^ (0.2)	8.2^d^ (0.2)
White index (WI)	—	65.3^a^ (0.5)	66.4^a^ (0.3)	67.1^a^ (0.3)	56.5^b^ (0.2)	54.2^b^ (0.2)
Specific volume	cm^3^/g	2.12^a^ (0.04)	1.04^b^ (0.03)	0.95^c^ (0.02)	1.01^b^ (0.03)	0.90^c^ (0.02)
Texture characteristics						
Crust hardness	N	6.1^d^ (0.5)	7.9^c^ (0.4)	10.2^b^ (0.2)	9.6^b^ (0.7)	13.5^a^ (0.2)
Cohesiveness	—	5.3^a^ (0.2)	4.4^b^ (0.1)	4.2^b^ (0.1)	3.1^c^ (0.1)	2.9^c^ (0.0)
Chewiness	N	6.5^d^ (0.2)	9.6^c^ (0.1)	10.2^bc^ (0.1)	11.1^b^ (0.3)	13.2^a^ (0.2)

Note

All values show the mean (standard deviation). ^a–d^Values within a row with different letters are significantly different (*p* < .05).

Abbreviations: CON, whole wheat flour; PLF10, 10 g of plantain flour/100 g; PLF20, 20 g of plantain flour/100 g; SF10, 10 g of soy flour/100 g; SF20, 20 g of soy flour/100 g.

The specific volume of composite bread samples was significantly decreased (*p* < .05) by substitutions of PLF and SF when compared to CON (Table 3). Compared with CON, the specific volume of bread samples with PLF20 and SF20 substitutions decreased by 55.2% and 57.5%, respectively. In general, the gluten network from wheat flour is responsible for the elasticity of the dough by trapping the carbon dioxide produced during fermentation. Thus, the lower specific volume in composite bread could be ascribed to the weakness of the gluten network due to dilution, reduction of hydration and gas retention ability, and interference with fibers and nongluten proteins (Sivam et al., 2010; Turfani et al., 2017). In accordance with other studies (Inyang & Asuquo, 2016; Islam et al., 2007; Taghdir et al., 2017), there was a reduction of specific loaf volume caused by addition of PLF, SF, and other nongluten flours. Inyang and Asuquo (2016) attributed a reduction in the specific volume and height of wheat–plantain composite bread to lower levels of the gluten network, and consequently, the dough was less able to rise due to weaker cell wall structure. When the gluten coagulates under the influence of heat during baking, it serves as a framework for the loaf which becomes relatively rigid. Aside from the effect of gluten dilution, Nilufer‐Erdil et al. (2012) and Shin et al. (2013) explained an increase of firmness and density in composite bread with SF substitution due to the formation of defects in the gluten from soy fiber, interchange of disulfide bonds between soy and gluten proteins, and absorption of water by soy fiber. Although the substitution of soluble fiber at low levels can enlarge the structure of dough and consequently improve the quality of bread (Sivam et al., 2011), excess of soy fiber could lead to a gluten dilution effect or gluten–fiber interaction (Kaack et al., 2006) which can reverse the formation of the gluten network (Ahmed et al., 2013). Furthermore, Ribotta et al. (2004) stated that protein aggregation and a corresponding loss of protein solubility in SF during baking can cause more firmness due to the lower specific volume of bread. However, the volume and pore formation during bread making are a complex process which is affected by composition as well as processing conditions such as mixing, proofing, and baking (Al‐Attabi et al., 2017). To improve dough stability, bread volume, and soft crumb texture of composite bread, hydrocolloids such as sodium alginate, *k*‐carrageenan, xanthan gum (Rosell et al., 2001), and hydroxypropylmethyl/cellulose can be used (Kim & Yokoyama, 2011; Sciarini et al., 2010) owing to their water absorption ability and gelling properties.

**FIGURE 2:**
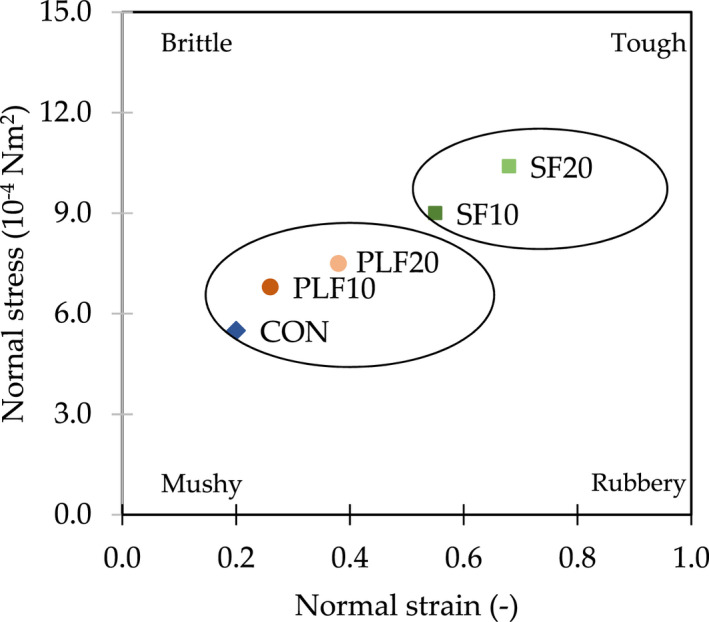
Texture map showing the distribution of textural attributes of control wheat bread and samples with partial substitution of plantain and soy flours. CON, whole wheat flour; PLF10, 10 g of plantain flour/100 g; PLF20, 20 g of plantain flour/100 g; SF10, 10 g of soy flour/100 g; SF20, 20 g of soy flour/100 g

From the textural characteristics of bread samples, a significant increase (*p* < .05) of crust hardness values was found in bread with PLF and SF substitution (Table 3), especially with increasing levels of replacement. Specifically, composite bread with SF20 and PLF20 substitution required more energy for the first fracture than the CON by 121.3% and 24.4%, respectively. On the other hand, substitution with PLF and SF caused a significant decrease (*p* < .05) in cohesiveness values. This result could be because the strength of the structure within the bread with PLF20 and SF20 substitutions was lower than in the CON sample by 20.8% and 45.3%, respectively. Partial replacement of composite breads with PLF and SF also significantly increased (*p* < .05) chewiness compared to CON, especially with the higher SF substitution. This indicated that more energy was required to chew the products to a ready state for swallowing. For example, the energy required to chew bread samples with part substitution by PLF20 and SF20 was 36.3% and 103.1%, respectively, higher than with CON. These textural characteristics are correlated with the loaf's specific volume as a lower specific volume result in a greater hardness because of the denser crumb and more compact cells (Sandri et al., 2017). As mentioned by Inyang and Asuquo (2016), increasing levels of substitution by unripe PLF led to a reduction of loaf volume and height of composite bread. Sciarini et al. (2012) explained that the disruption of soy protein/starch interaction negatively affects bread texture. A map of textural changes (Figure 2) also showed that substitution of PLF and SF affected the normal stress and normal strain values of breads. Samples in the texture map can be divided into two groups: CON and PLF vs. SF. Bread samples, especially SF10 and SF20, tended to be tough in textural characteristics, while control samples were much softer.

### 3.3 Consumer acceptance

**FIGURE 3:**
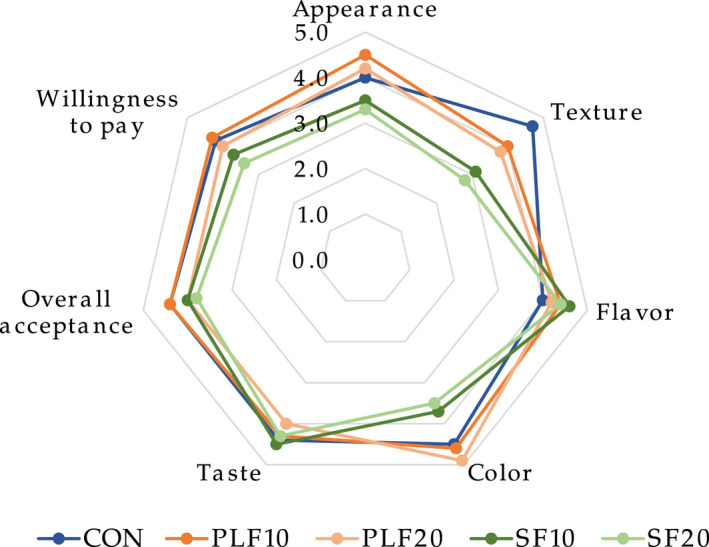
Changes in sensory attributes of control wheat bread and samples with plantain and soy flour replacement. All values show the mean (5 = *like extremely* and 1 = *dislike extremely*). CON, whole wheat flour; PLF10, 10 g of plantain flour/100 g; PLF20, 20 g of plantain flour/100 g; SF10, 10 g of soy flour/100 g; SF20, 20 g of soy flour/100 g

There were no significant differences (*p* > .05) in any organoleptic attributes between the wheat bread and composite bread samples with PLF10 and PLF20 substitution. The consumer acceptability results showed that composite bread with partial substitution of PLF had pronounced higher scores in appearance, flavor, and color characteristics as well as willingness to pay (Figure 3); significant lower scores of all characteristics, except for flavor and taste, were found (*p* < .05) in breads with partial substitution of SF. The sensory scores for all parameters, except color, obviously declined with the level of PLF substitution, while an increase of SF level led to a reduction in all sensory scores, particularly appearance, texture, and color. The results are consistent with Bank et al. (1997) who reported a lower color score in muffin made with partially defatted soy flour. In addition, Dhingra and Jood (2001) reported with an increase of SF at 15% and 20% levels of blending in bread resulted in lower scores for flavor and taste (Grewal, 1992). In contrast, Taghdir et al. (2017) showed that the highest total score of sensory evaluation was found in gluten‐free bread samples containing 15% SF, compared to 5% and 10% SF. Some studies have shown that incorporation of more than 15% SF in bread and biscuits did not produce acceptable bakery products (Awasthi et al., 2012; Farzana & Mohajan, 2015). Inyang and Asuquo (2016) indicated that approximately 20% of PLF can be substituted for wheat flour without any detrimental sensory effects. They also reported that a significant increase in the score for aroma was found in composite bread samples produced from the substitution of 30% to 50% of PLF.

### 3.4 Trade‐offs among physicochemical and sensory properties

**FIGURE 4:**
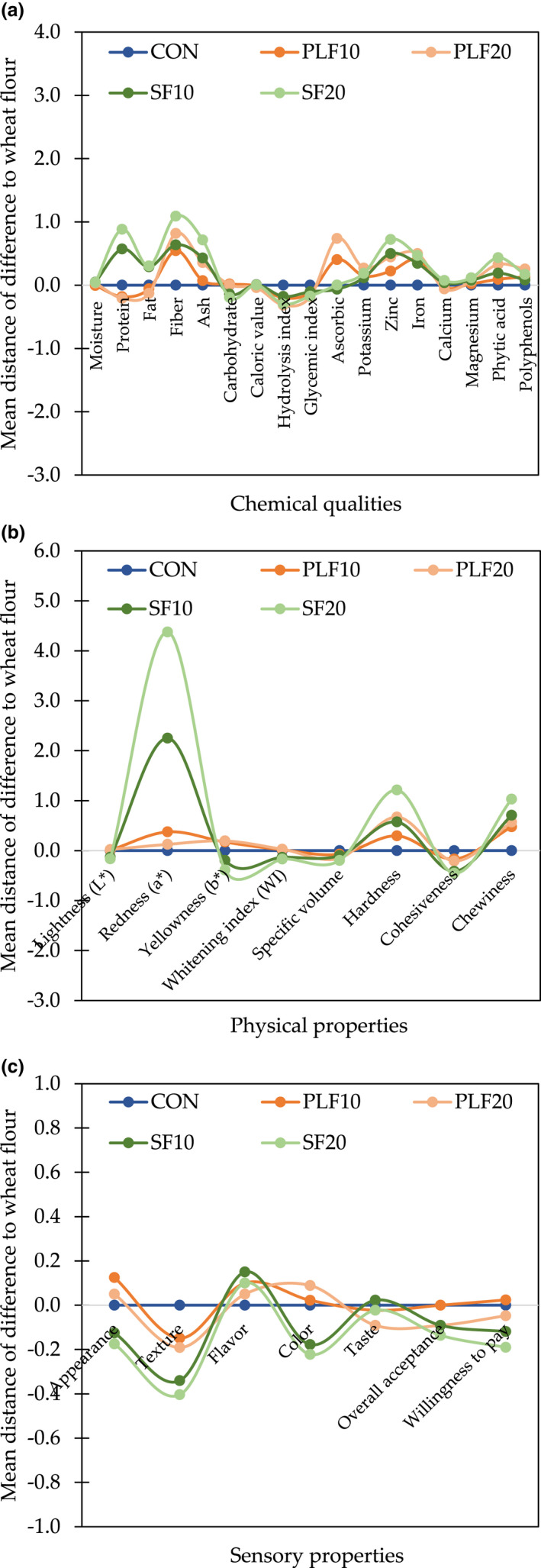
Trade‐offs among control wheat bread and different samples with substitution of plantain or soy flour: (a) chemical qualities; (b) physical properties; and (c) sensory properties. Distances represent the mean distance of each different substitute to wheat flour. CON, whole wheat flour; PLF10, 10 g of plantain flour/100 g; PLF20, 20 g of plantain flour/100 g; SF10, 10 g of soy flour/100 g; SF20, 20 g of soy flour/100 g

Nutritional quality, physical characteristics, and consumer acceptance have represented different trade‐off patterns among each category as well as across categories (Figure 4a–c). Breads with part substitution of PLF or SF have different nutritional attributes from CON, especially in fiber and mineral contents (Figure 4a). When physical properties are considered, the data did not indicate any clear patterns between PLF samples and CON, while SF expressed a large difference in a* and hardness values when compared with CON (Figure 4b). Although consumers showed a clear preference for PLF over CON and from CON to SF in appearance and color, textural characteristics of PLF and SF were negatively different from CON (Figure 4c).

## 4 CONCLUSIONS

This study provided data on nutrients, phytochemicals, mineral availability, physical property, and sensory acceptance of composite bread with partial replacement of wheat flour by PLF and SF. Although nutritional properties, especially fiber and mineral contents as well as GI value, of composite breads with substitution of PLF and SF significantly improved, the level of phytochemicals (antinutrients) should be addressed as it was found to influence the bioavailability of minerals, particularly in bread that is high in phytate but low in Zn and Fe contents. In addition, the substitution of PLF resulted in a brighter color than CON, while SF showed the opposite. The substitution of more PLF and SF up to a level of 20% also illustrated a lower specific volume and higher values of crust hardness and chewiness. Consumers accepted composite bread with substitution of PLF more in terms of appearance and color, while substitution of SF had a higher score in flavor and taste. Composite bread with PLF10 substitution received overall acceptability scores similar to those of CON wheat bread, while the lowest score of consumers' willingness to pay was found in SF20. This work shows that nutritional benefits and consumer preferences can represent trade‐offs in low‐income contexts. Although the partial substitution of PLF can meet the consumers' preferences, it will lead to low protein bread. Therefore, promotion of different substitutes in a low‐income context needs to consider specific attributes and the one‐size‐fits‐all approaches need to be avoided.

## CONFLICT OF INTEREST

The authors declare no conflict of interest.

## Data Availability

This manuscript has no associated data.
